# Early prediction of acute kidney injury after transapical and transaortic aortic valve implantation with urinary G1 cell cycle arrest biomarkers

**DOI:** 10.1186/s12871-016-0244-8

**Published:** 2016-09-08

**Authors:** Fabian Dusse, Michaela Edayadiyil-Dudásova, Matthias Thielmann, Daniel Wendt, Philipp Kahlert, Ender Demircioglu, Heinz Jakob, Simon T Schaefer, Kevin Pilarczyk

**Affiliations:** 1 Department of Thoracic and Cardiovascular Surgery, West German Heart and Vascular Center Essen, University Hospital Essen, Hufelandstraße 55, 45122 Essen, Germany; 2 Department of Anesthesiology and Intensive Care Medicine, Medical Center Cologne-Merheim, University of Witten/Herdecke, Ostmerheimerstrasse 200, 51109 Cologne, Germany; 3 Klinik für Anästhesiologie und Intensivmedizin, University Hospital Essen, Hufelandstraße 55, 45122 Essen, Germany; 4 Department of Cardiology, West German Heart and Vascular Center Essen, University Hospital Essen, Hufelandstraße 55, 45122 Essen, Germany; 5 Klinik für Anästhesiologie, Ludwig-Maximilians Universität München, Marchioninistraße 15, 81377 Munich, Germany; 6 Department of Intensive Care Medicine, Imland-Klinik Rendsburg, Lilienstraße 20-28, 24768 Rendsburg, Germany

**Keywords:** Acute kidney injury, AKI, TIMP-2, IGFBP7, Cell cycle arrest, NephroCheck, TAVI, TAVR, Biomarker

## Abstract

**Background:**

Acute kidney injury (AKI) is a common complication following transcatheter aortic valve implantation (TAVI) leading to increased mortality and morbidity. Urinary G1 cell cycle arrest proteins TIMP-2 and IGFBP7 have recently been suggested as sensitive biomarkers for early detection of AKI in critically ill patients. However, the precise role of urinary TIMP-2 and IGFBP7 in patients undergoing TAVI is unknown.

**Methods:**

In a prospective observational trial, 40 patients undergoing TAVI (either transaortic or transapical) were enrolled. Serial measurements of TIMP-2 and IGFBP7 were performed in the early post interventional course. The primary clinical endpoint was the occurrence of AKI stage 2/3 according to the KDIGO classification.

**Results:**

Now we show, that ROC analyses of [TIMP-2]*[IGFBP7] on day one after TAVI reveals a sensitivity of 100 % and a specificity of 90 % for predicting AKI 2/3 (AUC 0.971, 95 % CI 0.914-1.0, SE 0.0299, *p* = 0.001, cut-off 1.03). In contrast, preoperative and postoperative serum creatinine levels as well as glomerular filtration rate (GFR) and perioperative change in GFR did not show any association with the development of AKI. Furthermore, [TIMP-2]*[IGFBP7] remained stable in patients with AKI ≤1, but its levels increased significantly as early as 24 h after TAVI in patients who developed AKI 2/3 in the further course (4.77 ± 3.21 vs. 0.48 ± 0.68, *p* = 0.022). Mean patients age was 81.2 ± 5.6 years, 16 patients were male (40.0 %). 35 patients underwent transapical and five patients transaortic TAVI. 15 patients (37.5 %) developed any kind of AKI; eight patients (20 %) met the primary endpoint and seven patients required renal replacement therapy (RRT) within 72 h after surgery.

**Conclusion:**

Early elevation of urinary cell cycle arrest biomarkers after TAVI is associated with the development of postoperative AKI. [TIMP-2]*[IGFBP7] provides an excellent diagnostic accuracy in the prediction of AKI that is superior to that of serum creatinine.

## Background

Calcific aortic valve stenosis is the most common acquired valvular heart disease with the highest prevalence in the elderly. Despite the proven benefit of surgical valve replacement, almost 50 % of patients with indication for surgical therapy do not undergo open intervention mainly because of advanced age and significant comorbidities, resulting in high perioperative mortality [1]. Thus, transcatheter aortic valve implantation (TAVI) has become a valid option in patients with severe aortic stenosis who are i.e. considered ineligible for conventional surgical aortic valve replacement [2].

Acute kidney injury (AKI) following cardiac surgery is well investigated with a reported incidence of 4–30 %, and is associated with an increased mortality, which is proportional to the severity of AKI [3, 4]. Recently, the relevance of AKI after TAVI is increasingly recognized, as it seems to be a common complication with an incidence ranging between 12 and 57 % [5, 6]. The high prevalence of preoperative chronic kidney impairment, and intravascular hypovolemia as well as the use of contrast agents may contribute to the high incidence of AKI after TAVI. AKI has been shown to be an independent predictor of early mortality after TAVI and, subsequently the postoperative impairment of renal function has been defined as one of the major outcomes by the Valve Academic Research Consortium (VARC) [7]. Therefore, besides preoperative identification of patients at high risk for AKI, early detection of AKI seems to be reasonable. Although considered as a standard tool in clinical routine, serum-creatinine is not suitable for the early recognition of AKI due to inherent methodological problems [8]. New biomarkers detecting AKI, i.e. cystatin C or NGAL, showing superiority to creatinine in different patient populations, have either not been investigated in patients undergoing TAVI, or have shown poor accuracy in this setting [9]. More important, tissue inhibitor of metalloproteinases-2 (TIMP-2) and insulin-like growth factor binding protein 7 (IFGBP7) have recently been suggested as promising tools for the early detection of AKI in critically ill patients [10, 11]. Both proteins are inducers of the G1 cell cycle arrest, considered a key mechanism of AKI [12].

However, the utility of urinary cell cycle arrest biomarkers for the prediction of AKI in patients following TAVI is unknown. Therefore, it was the aim of the present study to describe the postoperative course of urinary [TIMP 2]*[IGFBP7] after TAVI and to determine whether this biomarkers are useful in predicting the development of AKI in this patient population.

## Methods

### Patients

**Fig. 1:**
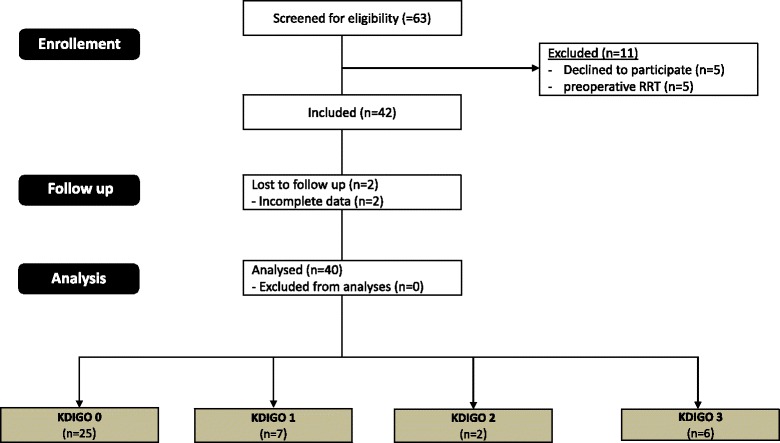
CONSORT 2010 Flow diagram. KDIGO = Kidney Disease: Improving Global Outcomes; RRT = renal replacement therapy

The study was conducted according to the principles of the Declaration of Helsinki and approved by the Institutional Ethic Review Board of the university Hospital Essen (approval date 01/09/2014, approval number: 13-5588-BO). Patients were included following written informed consent obtained from the patient or the patient’s guardian. We used the Standards for Reporting of Diagnostic Accuracy (STARD) statement for planning and conducting the study and preparation of the manuscript [13]. Sixty three consecutive patients were screened for study inclusion and finally 40 patients with severe symptomatic aortic stenosis who underwent transapical or transaortic TAVI at our institution were enrolled in this prospective study between January 2014 and September 2014. Twenty three patients were excluded as they refused to participate, incomplete data or other reasons as depicted in Fig. 1.

The indication for TAVI was discussed for each patient in an interdisciplinary consensus conference (“heart-team”) of cardiologists and cardiac surgeons. The patient’s or physician’s preference alone was not considered adequate for decision-making. Major outcomes were reported according to the standardized Valve Academic Research Consortium (VARC 2) criteria [7].

### Surgical procedure

All procedures were performed under general anesthesia with full hemodynamic monitoring in the hybrid laboratory under fluoroscopy and 3-dimensional (3D) transesophageal echo guidance (TEE). Hemodynamic monitoring included a five-lead electrocardiography system to monitor heart rate, rhythm and ST segments (leads II and V5), a pulse oximetry probe, continuous measurement of arterial pressure and blood sampling via the left radial artery, a triple-lumen central venous catheter and a 7.5-French balloon-tipped flow-directed pulmonary artery (PA) catheter (Becton Dickinson, Tense Belgium, Belgium) via the right internal jugular vein for continuous monitoring and measurement of PA and central venous pressures, intermittent cardiac output using thermodilution and measurement of blood temperature.

Sedation was performed using a continuous intravenous infusion of remifentanil (0.01–2 μg.kg − 1.min − 1) and titrated injections of midazolam (0.01–0.02 mg.kg − 1) as required, combined with local anesthesia (up to 400 mg lidocaine) of the groin.

General anesthesia was induced with fentanyl (2 μg.kg − 1) and etomidate (0.3 mg.kg − 1), followed by rocuronium (0.6 mg.kg − 1). Following tracheal intubation, patients’ lungs were mechanically ventilated with oxygen in air and anesthesia was maintained with inhaled isoflurane in end-tidal concentrations of 0.4–0.6 %, with additional intravenous fentanyl boluses of 2 μg.kg − 1.

Before the procedure, all patients received dual antiplatelet therapy with oral aspirin (100 mg) and clopidogrel (300 mg), and antibiotic prophylaxis with intravenous cefazolin (2 g). During the intervention, heparin (100–200 IU.kg − 1) was administered to elevate the activated clotting time to more than 250 s. After the procedure, all patients were transferred to the intensive care unit.

When mean PA pressure exceeded 30 mmHg and was associated with hemodynamic deterioration, nebulized iloprost (20 μg), intravenous milrinone (50 μg.kg − 1 over 10 min as a loading dose, followed by a continuous infusion of 0.4 μg.kg − 1.min − 1) or nitroglycerine (2–5 mg.h − 1) were administered, at the discretion of the responsible anesthetist. The transapical TAVI procedure was performed as previously described [14]. The Edwards SAPIEN™ aortic valve bioprosthesis was used in 13 patients whereas the Symetis™ valve was implanted in 15 patients.

Surgical approach for transaortic aortic valve implantation was achieved by mini J sternotomy or mini right thoracotomy in the 2^nd^ intercostal space, respectively. Detailed technical aspects of the transaortic TAVI procedure with the Symetis™ valve and the Ascendra II delivery system have been described in detail previously by Bapat et al. [15]. Device-specific medication consisted of acetylsalicylic acid 100 mg once daily for life and clopidogrel 75 mg daily for 6 months.

### Biomarker measurements

Urine samples for biomarker analysis were obtained within 4 h after surgery and then twice daily until discharge from the intensive/intermediate care unit and for a maximum of 4 days, whatever occurred first. Urinary concentration of TIMP-2 and IGFBP7 were measured with the commercially available and FDA-approved NephroCheck Test (Astute Medical, San Diego, CA, USA) - a point-of-care biomarker-test developed to measure the product of TIMP-2 and IGFBP7 urine concentration. Values were displayed as (ng/ml)^2^/1000. Serum creatinine was routinely measured prior and 4 h after surgery and at least twice daily in the post-operative period. Physicians in charge were blinded for TIMP-2*IGFBP7 urine concentrations, while laboratory investigators were blinded for clinical outcomes. Glomerular filtration rate (GFR) was estimated using the Modification of Diet in Renal Disease Study equation: GFR (ml/min/1.73 m^2^) = 175 × serum creatinine^-1.154^ × age^-0.203^ × (0.742 if female) × (1.212 if African American). ∆ eGFR was defined as change of estimated glomerular filtration rate (eGFR) at a specific time point from preoperative eGFR.

### Endpoint definition

AKI stage was determined daily on the basis of diuresis rate and serum creatinine concentration according to the Kidney Disease: Improving Global Outcomes classification (KDIGO) [16].

The primary endpoint was the new occurrence of acute kidney injury stage 2 or 3 within 48 h after surgery. This observation is crucial as prior studies had shown that AKI occurs within the first 24–72 h after TAVI in the majority of cases.

### Sample size calculation

The principle aim of this study was to investigate whether there is a significant difference between the mean [TIMP 2]*[IGFBP7] urine concentrations within the first 24 h after surgery in patients developing AKI 2/3 within 48 h after surgery compared to those who did not. Based on recently published data on [TIMP-2]*[IGFBP7] urine concentrations in adults undergoing cardiac surgery with cardiopulmonary bypass, we aimed to detect a difference of one unit in [TIMP-2]*[IGFBP7] urine concentrations with a standard deviation of 0.8 units [17]. The known and previously reported incidence of AKI following TAVI ranges between 11.7 % and 57 % [5, 6]. Performing a retrospective analysis of the last 30 patients undergoing TAVI at our institution, the incidence of KDIGO AKI stage 2 or 3 was 20 %, and this value was used for the sample size calculation. With a given probability of type I error (α) of 0.05, and a power (1 - β) of 0.8, the sample size calculation revealed a required minimum size of 40 patients in total.

### Statistical analysis

Statistical analyses were performed with SPSS Statistics 19 (IBM, Chicago, IL, USA). Continuous data were expressed as mean ± SD; categorical data were expressed as percentages. Comparisons between two groups were carried out using the unpaired Student’s *t*-test for normally distributed data and the Mann–Whitney *U* test for abnormally distributed data. Multiple groups were compared by means of one-way ANOVA. Univariate analysis was performed on the quantitative variables using the Student’s *t*-test or Mann–Whitney test and on the qualitative variables using the Chi^2^ test or Fisher’s exact test. Variables with *p* < 0.05 in the univariate analysis were included in a logistic regression model to determine the independent factors associated with death. Model calibration was tested using the Hosmer-Lemeshow test. Statistical significance was assumed for a *p*-value <0.05.

To measure the sensitivity and specificity of [TIMP 2]*[IGFBP7] urine concentrations at different cut-off values, a conventional receiver operating characteristic (ROC) curve was generated. The optimal cut-off level was defined by the highest Youden index (J = sensitivity + specificity – 1).

## Results

### Patients’ characteristics

Forty patients were included in this study (see Fig. 1). The procedural success rate was 100 %; no conversion to open aortic valve replacement (AVR) had to be done. The mean age of the patients was 81.2 ± 5.6 years, 16 patients were male (40.0 %). 35 patients underwent transapical, and five patients transaortic TAVI with equal distribution in study groups. AKI of any stage occurred in 15 patients (37.5 %): KDIGO AKI 1 was classified in seven patients (17.5 %) and KDIGO AKI 2 in two patients (5 %). Six patients suffered from KDIGO AKI 3 (15 %) and seven patients required RRT in the postoperative course (17.5 %).

The pre-procedural characteristics of patients undergoing TAVI suffering from AKI stage 2 or 3 according to KDIGO compared to those without significant renal impairment (KDIGO 0 or 1) are summarized in Table 1. Baseline demographics including age, gender, proportion of relevant comorbidities and technical details of surgery, e.g. length of surgery or applied contrast agent (AKI 2/3: [ml] 102.1 ± 35.8 vs. AKI 0/1: 131.0 ± 44.7, *p* = n.s.) were comparable between both groups. Incidence of preoperative atrial fibrillation was higher in patients suffering from postoperative KDIGO AKI 2/3. The severity of illness on the day of surgery assessed by Simplified Acute Physiology Score (SAPS) was higher in the group of patients with KDIGO AKI 2/3 (AKI 2/3: 33.8 ± 9.7 vs. AKI 0/1: 27.0 ± 6.7, *p* = 0.034). ICU stay tended to be longer in patients with moderate to severe renal impairment without reaching statistical significance. One patient in each study group died during the hospital stay (*p* = n.s.).

**Table 1:** Patient characteristics in patients with AKI 2/3 compared to patients with AKI ≤2

	AKI KDIGO 2/3	KDIGO 0/1	*p*-Value
	(*n* = 8)	(*n* = 32)	
Age [years]	81.4 ± 4.2	80.7 ± 5.9	n.s.
Male gender [n (%)]	3 (37.5)	13 (40.6)	n.s.
Weight [kg]	76.6 ± 13.6	70.2 ± 11.2	n.s.
Height [cm]	167.3 ± 6.6	164.7 ± 8.5	n.s.
Preoperative creatinine [mg/dl]	1.18 ± 0.26	1.12 ± 0.24	n.s.
Preoperative eGFR [ml/min/1.73 m^2^]	65.6 ± 15.9	65.9 ± 18.1	n.s.
Transapical approach [n (%)]	6 (75.0)	28 (87.5)	n.s.
SAPS	*33.8 ± 9.7*	*27.0 ± 6.7*	*0.034*
TISS	23.0 ± 4.5	22.6 ± 4.5	n.s.
LVEF [%]	49.3 ± 16.9	49.3 ± 10.9	n.s.
AVA [cm^2^]	0.83 ± 0.32	0.69 ± 0.22	n.s.
MPG [mmHg]	36.8 ± 23.5	42.9 ± 17.2	n.s.
Comorbidities [n (%)]			n.s.
Hypertension	7 (87.5)	24 (75.0)	n.s.
PHT	1 (12.5)	5 (15.6)	
Smoking	2 (25.0)	8 (25.0)	n.s.
Diabetes	3 (37.5)	10 (31.3)	n.s.
COPD	4 (50.0)	6 (18.8)	n.s.
Atrial fibrillation	7 (87.5)	13 (40.6)	*0.031*
Peripheral artery disease	1 (12.5)	11 (34.4)	n.s.
Recent myocardial infarction	2 (25.0)	2 (6.3)	n.s.
CAD	5 (62.5)	19 (59.4)	n.s.
Duration of surgery [h]	84.09 ± 0.03	84.06 ± 0.02	n.s.
Contrast agent [ml]	102.1 ± 35.8	131.0 ± 44.7	n.s.
Fluid balance on the day of surgery [ml]	2950. 0 ± 1053.8	2239.3 ± 954.6	n.s.
Cumulative catecholamine dosage on the day of surgery [μg/kgBW/min]	0.12 ± 0.09	0.06 ± 0.08	n.s
LCOS [n (%)]	1 (12.5)	0 (0)	n.s.
Sepsis [n (%)]	2 (25.0)	0 (0)	*0.03*
Septic shock [n (%)]	1 (12.5)	0 (0)	n.s.
LOS ICU/IMC [d]	8.3 ± 4.9	4.9 ± 3.8	n.s.
Outcomes According to VARC-2 [n (%)]			
30-d-mortality all cause	1 (12.5)	1 (3.1)	n.s.
30-d-mortality cardiovascular	1 (12.5)	0 (0)	n.s.
Stroke/TIA 30 d	0 (0)	1 (3.1)	n.s.
Bleeding			
Life-threatening/disabling	1 (12.5)	0 (0)	n.s.
Major	0 (0)	0 (0)	n.s.
Minor	0 (0)	0 (0)	n.s.
Unplanned use of CPB	0 (0)	0 (0)	n.s.
Conversion to open surgery	0 (0)	0 (0)	n.s.
Pacemaker implantation	1 (12.5)	0 (0)	n.s.
Myocardial infarction	0 (0)	0 (0)	n.s.
Procedure success	8 (100)	32 (100)	n.s.
Access site complications			
minor	0 (0)	0 (0)	n.s.
major	0 (0)	0 (0)	n.s.

*COPD* chronic obstructive pulmonary disease, *SAPS* Simplified Acute Physiology Score, *TISS* Therapeutic Intervention Scoring System, *eGFR* estimated glomerular filtration rate, *ICU* intensive care unit, *IMC* intermediate care unit, *LVEF* left ventricular ejection fraction, *PHT* pulmonary hypertension, *CAD* coronary artery disease, *LCOS* Low cardiac output syndrome, *LOS* length of stay, *VARC* Valve Academic Research Consortium

The incidence of sepsis was higher in the group of patients with AKI 2/3 (2/8 (25 %) vs. 0/32 (0 %), *p* = 0.03). Preoperative estimated glomerular filtration rate (eGFR) and serum creatinine were not associated with the development of postoperative AKI 2/3 (preoperative eGFR: AKI 2/3 65.6 ± 15.9 vs. AKI 0/1 65.0 ± 18.1 ml/kg/1.73 m^2^; preoperative serum creatinine: AKI 2/3 1.18 ± 0.26 vs. AKI 0/1 1.12 ± 0.24 mg/dl).

### Perioperative course of biomarkers

In patients developing AKI 2/3 within the next 48 h, [TIMP-2]*[IGFBP7] increased significantly already on the 1^st^ postoperative day (4.62 ± 3.14 (ng/ml)2/1000) followed by a decline to 3.40 ± 1.86 on postoperative day (POD) 2. In the further course [TIMP-2]*[IGFBP7] increased to a maximum of 27.09 ± 36.74 (ng/ml)^2^/1000 on the ^4th^ day.

No significant rise in urinary [TIMP-2]*[IGFBP7] urine concentration was observed in patients with KDIGO AKI 0/1 at any time with a maximum value on the 3th postoperative day of 1.75 ± 3.63 indicating that surgical intervention with TAVI per se has no influence on the investigated G1 cell cycle arrest biomarkers.

**Fig. 2:**
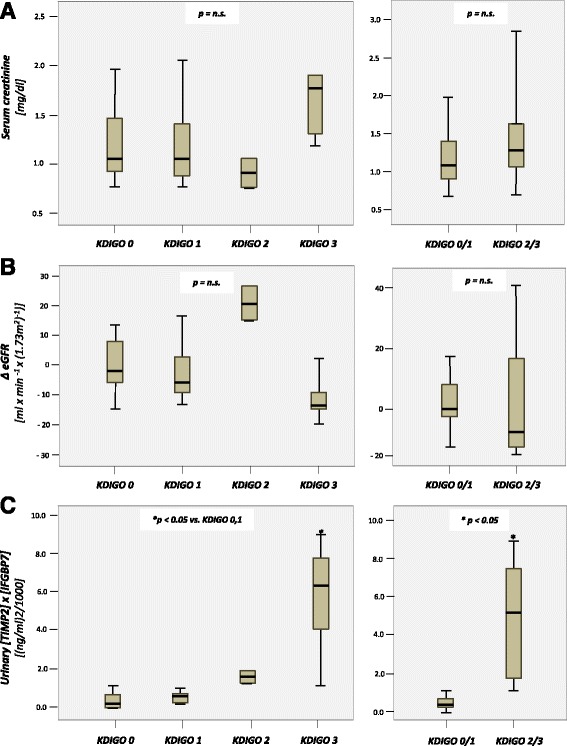
Postoperative course of serum creatinine **a**, eGFR **b** and [TIMP-2]*[IGFBP7] **c** in patients with AKI ≥ 2 compared to patients with AKI ≤ 1. * = *p* < 0.05 between patients with AKI ≥ 2 and patients with AKI ≤1

In patients with KDIGO AKI 0/1, serum creatinine as well as eGFR remained stable at all times with no significant undulations over time. In contrast, elevated serum creatinine levels could be observed on POD 2 with a maximum of 1.64 ± 0.99 mg/dl. Accordingly, eGFR showed a significant fall on POD 2 with 38.86 ± 23.79 ml/min/1.73 m^2^. Furthermore, the postoperative courses of serum creatinine, eGFR and urinary [TIMP 2]*[IGFBP7] urine concentrations for patients with AKI stage 2/3 and for those with no or mild AKI (KDIGO stage 0–1) are displayed in Fig. 2.

**Fig. 3:**
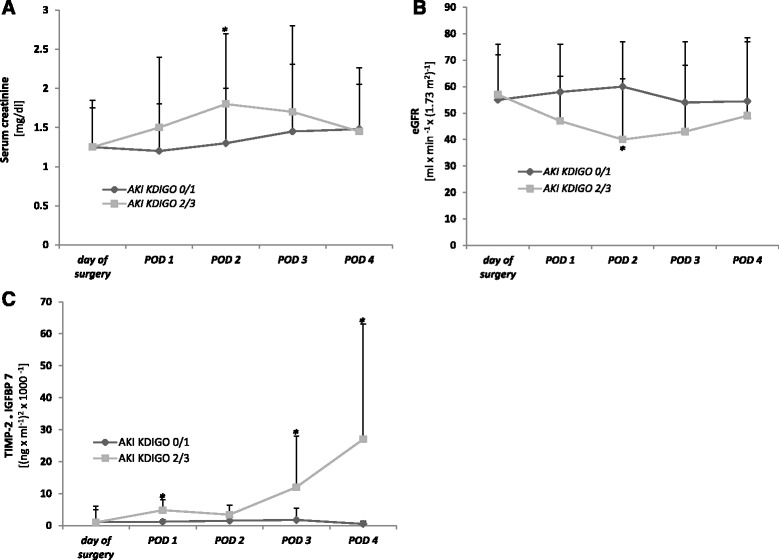
Boxplots of biomarker values and ∆ eGFR on postoperative day 1 after TAVI grouped by KDIGO stage. **a** Serum creatinine. **b** ∆ eGFR. **c** Urinary [TIMP 2]*[IGFBP7]

Distribution of biomarkers and eGFR measured on POD 1 following TAVI according to the KDIGO classification is shown in Fig. 3. [TIMP 2]*[IGFBP7] urine concentrations in patients with KDIGO AKI 3 (median 6.49, range 1.06–8.66) were significantly higher than in patients with KDIGO AKI 0 (median 0.21, range 0.06–3.03) or KDIGO AKI 1 (median 0.35, range 0.06–0.57). Accordingly, patients with AKI 2 or 3 showed significantly higher [TIMP 2]*[IGFBP7] urine concentrations than patients with AKI 0–1 (median 5.11, range 1.06–8.66 vs. 0.23, range 0.06–3.03, *p* < 0.05).

In contrast, neither serum creatinine concentrations nor eGFR differ significantly in subgroups stratified by KDIGO.

### Prediction of AKI with biomarkers

Using ROC analyses, serum creatinine concentrations measured preoperatively (AUC 0.458, 95 % CI 0.226–0.691, SE 0.119, *p* = 0.720) as well as 4 h after intervention (AUC 0.500, 95 % CI 0.186–0.814, SE 0.160, *p* = 1.00) turned out to be a bad predictor for AKI 2/3. The same applies for eGFR measured before surgery as well as the change of eGFR from preoperative to 4 h after intervention.

In contrast, diagnostic accuracy for [TIMP 2]*[IGFBP7] 4 h after TAVI was better with a sensitivity of 75 % and a specificity of 55.6 % when using a cut-off value of 0.185 (AUC 0.646, 95 % CI 0.375–0.916, SE 0.138, *p* = 0.312). [TIMP 2]*[IGFBP7] measured on day one after TAVI showed a sensitivity of 100 % and a specificity for predicting KDIGO AKI 2/3 (AUC 0.971, 95 % CI 0.914–1.000, SE 0.0299, *p* = 0.001, cut off 1.03). Compared to [TIMP 2]*[IGFBP7] urine concentrations, serum creatinine concentrations on day one after surgery as well as the change in GFR from preoperative baseline to POD 1 yielded low predictive capacities for AKI 2/3 (serum creatinine: AUC 0.629, 95 % CI 0.438–0.870, SE 0.11, *p* = 0.189, cut off 1.37 mg/dl, sensitivity 50 %, specificity 82.1 %; ∆ GFR: AUC 0.586, 95 % CI 0.359–0.944, SE 0.149, *p* = 0.196, cut off 7 ml/min/1.73 m^2^, sensitivity 62.5 %, specificity 82.1 %;).

**Fig. 4:**
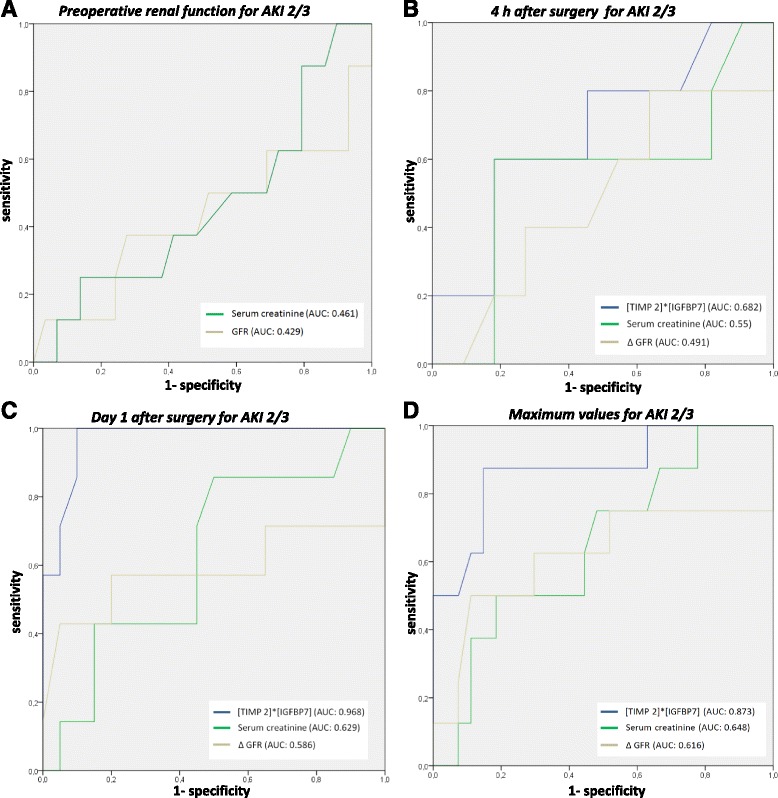
Receiver operator characteristic (ROC) curve for the prediction of AKI 2/3. **a** Preoperative serum creatinine and eGFR. **b** [TIMP 2]*[IGFBP7], serum creatinine and ∆ eGFR 4 h after TAVI. **c** [TIMP 2]*[IGFBP7],serum creatinine and ∆ eGFR on postoperative day 1 after TAVI. **d** Maximum [TIMP 2]*[IGFBP7], serum creatinine and ∆ eGFR during the first 24 h after surgery. AKI = acute kidney injury, AUC = Area under the curve, IGFBP7 = insulin-like growth factor binding protein 7, KDIGO = Kidney Disease: Improving Global Outcomes; ROC = Receiver operating characteristic; RRT = renal replacement therapy, TIMP- 2 = tissue metalloproteinase 2. * defined as maximum level in the first 24 h after surgery

At a cut-off of 0.91, the maximum value of [TIMP 2] *[IGFBP7] urine concentrations within 24 h after intervention showed a 87.5 % sensitivity and a 82.8 % specificity for detecting patients who develop KDIGO AKI 2/3 (AUC 0.869, SE 0.080, 95 % CI 0.721–1.0, *p* = 0.002). The diagnostic accuracy for [TIMP 2] *[IGFBP7] was better than for maximum serum creatinine concentrations within the first 24 h after intervention with a sensitivity of 75.0 % and a specificity of 55.2 % for a cut-off-value 1.160 (AUC 0.655, SE 0.108, *p* = 0.184, 95 % CI 0.443–0.867) as well as maximum ∆ GFR (AUC 0.616, SE 0.142, 95%CI 0.338–0.983, *p* = 0.326). The concordant ROC curves are illustrated in Fig. 4.

In addition, the maximum value of [TIMP 2]*[IGFBP7] within 24 h after intervention showed a sensitivity 100 % and a specificity of 80.0 % for detecting patients with need for renal replacement within the next 72 h (AUC 0.919, SE 0.048, 05 % CI 0.824–1.0, *p* = 0.001). Applying previously published cut-off points (0.3 and 2.0 (ng/ml)^2^/1000) showed either low sensitivity or low specificity (Table 2).

**Table 2:** Analysis of applying best and previously published cutoff points of urinary [TIMP 2]*[IGFBP7] for the prediction of AKI KDIGO 2/3

	AUC	Cut-Off	Sensitivity	Specificity	Youden’s index
4 h after surgery	0.646	0.185	0.75	0.56	0.31
		0.3	0.38	0.67	0.05
		2.0	0.13	1.0	0.13
Day 1 after surgery	0.971	1.0	1.0	0.9	0.9
		0.3	1.0	0.55	0.55
		2.0	0.67	0.95	0.62
Maximum early value^a^	0.869	0.91	0.88	0.83	0.71
		0.3	0.88	0.48	0.36
		2.0	0.5	0.93	0.43

*AKI* acute kidney injury, *AUC* Area under the curve, *IGFBP7* insulin-like growth factor binding protein 7, *KDIGO* Kidney Disease: Improving Global Outcomes, *RRT* renal replacement therapy, *TIMP-2* tissue metalloproteinase 2

^a^defined as maximum level in the first 24 h after surgery

## Discussion

In our prospective observational trial of 40 patients undergoing transapical or transaortic TAVI the elevation of urinary [TIMP 2]*[IGFBP7] concentrations within 24 h after surgery is associated with the onset of postoperative AKI within the next 72 h. [TIMP 2]*[IGFBP7] urine concentrations show an excellent diagnostic accuracy for the prediction of severe AKI requiring RRT. In contrast, neither serum creatinine concentrations (preoperative, postoperative) nor changes of GFR were able to predict the occurrence of AKI reliably. Our results clearly demonstrate that [TIMP 2]*[IGFBP7] can be used to identify patients at high risk for AKI.

Incidence, mechanisms and outcome of renal impairment after TAVI are not well investigated. According to a reported probability of 12 to 57 % for the development of an AKI after TAVI, the risk of renal impairment is very high, despite avoidance of cardiopulmonary bypass and major surgical trauma occurring during open aortic valve replacement [5, 6]. Intraprocedural episodes of hypotension, intraoperative emboli, occurrence of paravalvular aortic regurgitation with a reduction in diastolic renal blood flow and the use of contrast agents are some of the factors potentially contributing to this high incidence of AKI after TAVI. A recent meta-analysis including 25 studies with a total of 8874 patients identified KDIGO AKI ≥2 as the strongest predictor for 30-d-mortality (OR 18.0, 95 % CI 6.3 to 52) whereas KDIGO AKI 3 was an important determinant of midterm mortality after TAVI [18].

The current assessment of renal function by means of serum creatinine or urine output has a number of major limitations: creatinine is insensitive to small but significant declines in GFR, and there is regularly a delay between more extensive kidney injury and the subsequent rise of serum creatinine. In addition, numerous non-renal factors such as age, gender, muscle mass or liver disorders may be associated with decreased creatinine production or the dilution of serum creatinine concentrations [8]. New biomarkers suggested for an accurate and earlier detection of AKI are various, including cystatin C, neutrophil gelatinase-associated lipocalin (NGAL), kidney injury molecule-1 (KIM-1), liver-type fatty acid binding protein (L-FABP), etc. Among these, only cystatin C and NGAL seem to be reliable in selected patient cohorts and automated assay methods are commercially available.

However, in a recently published study, urinary NGAL levels in 34 consecutive patients measured 4 h after transfemoral TAVI were comparable between patients with AKI and those without (41.6 ± 35.7 vs. 45.3 ± 38.7 ng/mL, *p* = 0.57) [19]. In a prospective study enrolling 68 consecutive patients with symptomatic severe aortic stenosis undergoing transfemoral or transapical TAVI, cystatin C levels in patients developing AKI were comparable to those with normal postoperative renal function within the first two postoperative days. Thus, Cystatin C does not represent a stand-alone biomarker for the early detection of AKI after TAVI [9]. Other authors confirmed these findings and could not detect any prognostic impact of cystatin C in this patient population [20]. Consequently, due to very limited evidence in the TAVI population and inconsistent results from recently published studies, the prognostic impact of NGAL and Cystatin C in this clinical setting is more than disputable [9, 19]. Currently there is still no valid biomarker available for the early and reliable detection of AKI in critically ill patients in general and in the TAVI population in particular. Thus measurement of [TIMP 2]*[IGFBP7] urine concentrations as early as 24 h following intervention might be a promising approach even in patients undergoing minimal invasive TAVI procedures, as we have shown in our study.

In detail, recently a total of 340 potential biomarkers had been investigated in the urine and serum for the prediction of AKI of 744 adult critically ill patients [21]. The two cell cycle arrest proteins TIMP-2 and IGFBP7 were identified to have better diagnostic accuracy than any other biomarker. Furthermore, in the SAPPHIRE study, TIMP-2 and IGFBP7 exhibited an AUC of 0.76 and 0.79 AUC for the development of AKI (KDIGO stage 2 or 3) within 12 h, respectively. The combination of both biomarkers increased the AUC to 0.80 [21]. The prospective OPAL study enrolling 154 critically ill adults from six sites in the USA used two clinical cut-off values for urinary [TIMP-2]*[IGFBP7] based on the data from the SAPPHIRE cohort: A low (0.3) and a high (2.0) cut-off value were chosen to achieve either a high sensitivity or a high specificity to identify patients who are at high risk for AKI, respectively [10]. For the 0.3 cut-off, sensitivity was 89 % and specificity of 53 %. For 2.0, sensitivity was 42 % and specificity 95 %. The prospective multicenter TOPAZ study enrolled a heterogeneous population of 420 patients admitted to the ICU to validate the utility of [TIMP-2]*[IGFBP7] at a high-sensitivity pre-specified cut-off greater than 0.3 for the AKI defined as KDIGO stage 2 or 3 within 12 h [11]. Secondary analysis was performed for a cut-off value of 2.0. Sensitivity of [TIMP-2]*[IGFBP7] was 92 % with a negative likelihood ratio of 0.18. Patients with urinary [TIMP-2]*[IGFBP7] greater than 0.3 had seven times the risk for AKI compared to those critically ill patients with a test result below 0.3.

In summary, we now show that the combination of the two biomarkers TIMP2 and IGFBP7 seems to be highly predictive to identify patients who will develop moderate to severe AKI in the next 12–24 h following TAVI.

Both biomarkers - TIMP-2 and IGFBP7- are inducers of G1 cell-cycle arrest, considered as a key mechanism of AKI [12]. Tissue inhibitors of metalloproteinases (TIMPs) are endogenous inhibitors of matrix metalloproteinases (MMPs), a large family of zinc endopeptidases having important roles in extracellular matrix (ECM) remodeling. Four TIMPs (TIMP1–4) have been identified as inhibitors of 26 MMPs.

IGFBP-7 is a secreted glycoprotein that binds to insulin growth factors. According to the Human Protein Atlas, IGFBP-7 is expressed in the glomeruli and tubules of healthy humans. IGFBP-7 plays a pivotal role in G1 cell-cycle arrest. Renal tubular cells can enter a short period of G1 cell-cycle arrest following injury from experimental sepsis or ischemia. This process may prevent cells from dividing when DNA is damaged and may represent an early response to renal injury [12].

Studies investigating the role of [TIMP 2]*[IGFBP7] in the prediction of AKI and defining cut-off values in cardiovascular patient populations are rare and currently restricted to adult and pediatric open cardiac surgery [17]. Meersch et al. were able to show that the maximum [TIMP 2]*[IGFBP7] concentration within the first 24 h after cardiac surgery has a sensitivity of 0.92 and specificity of 0.81 at a cut-off value of 0.50 for the prediction of AKI, performing better than creatinine or NGAL [22] in 50 patients. Moreover, the decline in urinary [TIMP 2]*[IGFBP7] concentrations was a strong predictor for renal recovery before hospital discharge. The same working group investigated the utility of [TIMP 2]*[IGFBP7] in 51 children undergoing open cardiac surgery showing significantly higher [TIMP 2]*[IGFBP7] levels 4 h after surgery in comparison to children who did not develop AKI. [TIMP 2]*[IGFBP7] measured 4 h after intervention demonstrated a sensitivity of 0.83 with a specificity of 0.77 (AUC 0.85, cut-off value 0.70) [17].

As etiologies and pathophysiology of AKI differ substantially between patient populations, cut-off values from septic or medical patients might not be applicable to patients undergoing TAVI. Accordingly, we defined cut-off values for interventional TAVI procedures in our study which were different from recently published studies: In the Discovery, Sapphire and Opal study, best cut-off values for discrimination of patients with high risk for developing AKI in the future was 0.3 (ng/ml)^2^/1000, whereas Meersch set the value of 0.5 (ng/ml)^2^/1000 for the adult and 0.70 (ng/ml)^2^/1000 for the pediatric cardiothoracic population.

In contrast to serum creatinine, the postoperative course of the investigated cell cycle arrest biomarkers showed a characteristic biphasic pattern: serum creatinine rises slowly being significantly higher in the AKI group on postoperative day 2, still rising on POD 3 and staying stable on POD 4. Unlike, [TIMP 2]*[IGFBP7] rises as early as 24 h after surgery and then already falls on POD 2. In the further course [TIMP 2]*[IGFBP7] increases again on POD 3 and 4.

TIMP-2 and IGFBP7 have been implicated in the G1 cell-cycle arrest phase representing the earliest point of cellular stress. As TIMP-2 and IGFBP7 increase in response to a wide variety of insults (inflammation, oxidative stress, drugs and toxins) very fast, this biphasic course may implicate that besides surgery with application of contrast agent being responsible for the first peak of the graph, further insults, e.g. hemodynamic disturbances, may have led to the second peak on POD 3 whereas serum creatinine is not able to detect and discriminate these multi hits to the kidney remaining high from POD 1 to POD 3.

The costs for the NephroCheck Test are approximately 35 €. This is significantly higher than the costs for the gold standard – serum creatinine. However, a recently published study could show that the presence of AKI in 891 critically ill patients with ARDS, MI and sepsis resulted in additional costs of 2,019,120.42 € at the University Hospital of Regensburg in 2013 translating into additional costs of 2266 € per patient [23]. The authors conclude that early recognition of patients at risk may be one important step to decrease the incidence and severity of AKI and save costs for the national healthcare system. Therefore, it might be hypothesized that the higher costs for the measurement of the urinary G1 cell cycle arrest biomarkers might be at least partly be compensated by the decreased therapy costs for these patients.

Difficulties in prediction and early identification of AKI have hindered the ability to develop preventive and therapeutic interventions. If recognized earlier, nephroprotective measures could be considered to reduce exposure to renal insults and potentially avoid the development of higher stage AKI. Although there are no specific therapies for AKI, the optimization of fluid balance and hemodynamics as well as a medication review with avoidance of nephrotoxic drugs can reduce the incidence and severity of AKI and improve long-term outcomes [24]. In addition, earlier commencement of RRT in critically ill patients with incipient AKI may be beneficial with a reduction of mortality [25].

### Limitations

There were some limitations to our study. Due to the relatively small sample size, the predicted cut-off values for [TIMP-2]*[IGFBP7] concentrations in the urine may be different from those obtained in other populations. In addition, the number of patients with AKI I small and consecutively the AUC 95 % CIs are very broad. Thus, interpretation of the data should be performed with caution. In the present study, we only investigated the commercially available test kit measuring the product of [TIMP-2]*[IGFBP7]. Therefore, we cannot draw any conclusions about the prognostic properties of TIMP-2 and IGFBP7 alone in comparison to their multiplication. Diuresis is difficult to interpret in early postoperative care after cardiac surgery, as decreased urinary output depends on the amount of intravenous fluid administered as well as the application of a diuretic to avoid volume overload in patients with restricted cardiac function. This may blur the clinical effect of AKI.

## Conclusion

[TIMP 2]*[IGFBP7] values of 1.0 or greater on day one after interventional TAVI identify patients at high risk for developing KDIGO AKI stage 2/3. Therefore, [TIMP 2]*[IGFBP7] can be considered a useful tool in the early prediction of AKI in patients after TAVI. However, future studies are mandatory to verify whether [TIMP 2]*[IGFBP7] guided therapy can prevent severe AKI and reduce morbidity and mortality.

## Abbreviations

AKI: Acute kidney injury
AUC: Area under the curve
BMI: Body mass index
CAD: Coronary artery disease
CBP: Cardiopulmonary bypass
COPD: Chronic obstructive pulmonary disease
eGFR: estimated glomerular filtration rate
ICU: Intensive care unit
IGFBP7: Insulin-like growth factor binding protein 7
KDIGO: Kidney Disease: Improving Global Outcomes
LCOS: Low cardiac output syndrome
log ES I: logistic EuroScore I
LOS: Length of stay
LVEF: Left ventricular ejection fraction
PHT: Pulmonary hypertension
ROC: Receiver operating characteristic
RRT: Renal replacement therapy
SAPS: Simplified Acute Physiology Score
TIMP- 2: Tissue metalloproteinases 2
TISS: Therapeutic Intervention Scoring System
VARC: Valve Academic Research Consortium.
