# Erratum to: ‘Early prediction of acute kidney injury after transapical and transaortic aortic valve implantation with urinary G1 cell cycle arrest biomarkers’

**DOI:** 10.1186/s12871-016-0249-3

**Published:** 2016-10-03

**Authors:** Fabian Dusse, Michaela Edayadiyil-Dudásova, Matthias Thielmann, Daniel Wendt, Philipp Kahlert, Ender Demircioglu, Heinz Jakob, Simon T. Schaefer, Kevin Pilarczyk

**Affiliations:** 1 Department of Thoracic and Cardiovascular Surgery, West German Heart and Vascular Center Essen, University Hospital Essen, Essen, Germany; 2 Department of Anesthesiology and Intensive Care Medicine, Medical Center Cologne-Merheim, University of Witten/Herdecke, Cologne, Germany; 3 Klinik für Anästhesiologie und Intensivmedizin, University Hospital Essen, Essen, Germany; 4 Department of Cardiology, West German Heart and Vascular Center Essen, University Hospital Essen, Essen, Germany; 5 Klinik für Anästhesiologie, Ludwig-Maximilians Universität München, Munich, Germany; 6 Department of Intensive Care Medicine, Imland-Klinik Rendsburg, Rendsburg, Germany

Unfortunately, the original version of this article [1] contained an error. Figure 2 and Figure 3 have been swapped with each other. Figure 2 is supposed to be the graph and figure 3 is supposed to be the boxplot. There will also be an update to correct this.
